# Recombinant human relaxin versus placebo for cervical ripening: a double-blind randomised trial in pregnant women scheduled for induction of labour

**DOI:** 10.1186/s12884-016-1046-1

**Published:** 2016-09-05

**Authors:** Gerson Weiss, Sam Teichman, Dennis Stewart, David Nader, Susan Wood, Peter Breining, Elaine Unemori

**Affiliations:** 1 Department of Obstetrics, Gynecology and Women’s Health, New Jersey Medical School, Rutgers, The State University of New Jersey, Newark, NJ 07101 USA; 2 At the time of study conduct, an employee of Corthera, Inc. (formerly BAS Medical), 1660 South Amphlett Blvd., San Mateo, CA 94402 USA

**Keywords:** Cervical ripening, Relaxin, Serelaxin

## Abstract

**Background:**

Nonclinical studies indicate that the hormone relaxin is a good candidate for a safe cervical ripening agent that does not cause uterine contractions.

**Methods:**

This Phase II study (conducted November 2, 2005–October 20, 2006) was a randomised, double blind, placebo controlled trial testing 24-h intravenous infusion of serelaxin (recombinant human relaxin) or placebo for cervical ripening in 72 healthy, primiparous women. Eligible subjects had a singleton pregnancy ≥40 weeks, were planned for elective induction, had vertex presentation of the fetus, intact membranes and a Bishop score at screening ≤4. In Part A of the study, safety evaluation of three escalating doses of serelaxin (7.5, 25 or 75 μg/kg/day) or placebo was performed in 22 subjects admitted to the hospital 24 h prior to scheduled induction (*n* = 7, 4, 4, and 7 subjects, respectively). The highest safe dose from Part A and placebo were then tested in Part B for safety and cervical ripening (*n* = 25 subjects/arm). Planned randomisation ratio was of 4:2 (serelaxin:placebo) for each dose group in Part A and 1:1 for Part B. For analysis, subjects in Part B were pooled with those receiving the same dose in Part A and all subjects receiving placebo were pooled. The primary efficacy endpoint was change from baseline in Bishop score at 6, 12 and 24 h or end of study drug administration. Maternal safety evaluations included adverse events and vital signs through 4 weeks. Fetal assessments included serial heart rate monitoring and nonstress testing. Neonatal assessments included Apgar scores, NICU admissions, and adverse events through 4 weeks.

**Results:**

Overall, 74 subjects were randomized and 72 were treated. There were no significant differences between the groups receiving the highest safe dose of serelaxin (75 μg/kg/day) and placebo in the primary or secondary efficacy endpoints. Changes from baseline in Bishop score at 24 h were 4.19 ± 1.9 and 3.26 ± 2.26 in the pooled placebo and serelaxin groups, respectively (*p* = 0.2507). Serelaxin was well tolerated and no anti-serelaxin antibodies were detected in either subjects or neonates.

**Conclusion:**

Serelaxin infusion at the end of pregnancy was well tolerated but did not advance cervical ripening.

**Trial registration:**

Clinicaltrials.gov identifier NCT00259103 (15 November 2005).

## Background

Induction of labour is one of the most common procedures in obstetrics, being performed in approximately 25 % of women in industrialized countries [1]. In women with a high degree of cervical ripeness, induction of labour can usually be achieved with simple types of intervention [2]. Conversely, if the cervix is not ripe, induction of labour is much more likely to fail. In order to facilitate cervical softening, thinning and dilation for the purpose of enabling labour induction, both mechanical and pharmacologic ripening agents are utilized [2]. Prostaglandins are the most commonly used pharmacological aid and are effective in cervical preparation but are also associated with an increased risk of uterine hyperstimulation, potentially leading to fetal heart rate changes [2–4]. Estrogens, nitric oxide donors, and hyaluronidase have also been postulated as potential cervical ripening agents but currently, none are recommended for use in this regard [2, 4]. Therefore, there is still a need for a cervical ripening agent that does not cause uterine contractions.

The naturally occurring hormone, relaxin, which mediates the hemodynamic changes that occur during pregnancy [5], causes cervical ripening in rodents and pigs [6]. In these species, serum relaxin is present from mid-pregnancy and increases markedly at the end of pregnancy in an “antepartum surge” that contributes to cervical ripening. Blocking relaxin activity using anti-relaxin neutralizing antibodies at this time inhibits cervical ripening in rats [7]. Relaxin- or relaxin receptor gene-deficient mice demonstrate an impaired ability to deliver pups, largely due to defective cervical ripening [8, 9]. In mice, relaxin causes a reduction in the density of collagen fibers [10], an alteration in aquaporin expression and an increase in hyaluronic acid content in the cervix [11]. These changes are associated with softening and increased distensibility of the cervix [12].

Although reports regarding the association between endogenous circulating relaxin levels and cervical ripening in women have generally shown a lack of correlation [13–17], relaxin binding sites are detected in the human cervix [18] and relaxin increases the expression of matrix metalloproteinases and glycosaminoglycans in cultured human cervical cells [19].

For these reasons, whether relaxin can cause cervical ripening has long been of clinical interest [20]. Multiple clinical trials have been conducted in the past to assess the ability of relaxin to enhance cervical ripening in a clinical setting [20–24]. Of these, a Cochrane review [25] included 4 blinded, randomized studies that tested intracervically and intravaginally administered purified porcine or recombinant human relaxin versus placebo and concluded that the data in these studies were not sufficient to recommend using relaxin to enhance labour or ripen the cervix. Pharmacokinetic analysis of intracervical and intravaginal application of serelaxin indicated that absorption via these topical routes is limited [26], suggesting that inadequate dosing may have played a role in the observed lack of efficacy. Therefore, whether relaxin can indeed ripen the cervix in women remained an open question.

We report the results of a completed clinical trial, which is listed in the Cochrane review as awaiting results [25] and tested intravenous (IV) administration of recombinant human relaxin (termed “serelaxin,” the international nonproprietary name) versus placebo for the ability to ripen the cervix in late pregnant women. The specific trial objectives were: (1) to test the safety of 24-h IV serelaxin at pharmacological levels in pregnant women at ≥40 weeks’ gestation, and (2) to determine whether this mode of dosing could ripen the cervix and induce labour in these women.

## Methods

This study was a prospective, randomized, double-blind, placebo-controlled pilot Phase II trial evaluating serelaxin for safety and efficacy in women ≥40 weeks gestation admitted for elective induction of labour (clinicaltrials.gov identifier NCT00259103). It was conducted at 12 clinical sites, including teaching and community hospitals, in Russia between November 2, 2005 and October 20, 2006 in compliance with the protocol, International Conference on Harmonization, Good Clinical Practices, Ministry of Public Health and Social Development of the Russian Federation, local regulations and the standard operating procedures of the study sponsor, Corthera, Inc. (formerly BAS Medical). The protocol and informed consent were approved by the governing Ethics Committee at each study site and the Russian Federal Body for the Control of Pharmaceutical Agents. Subjects were screened for eligibility in approved clinics and hospitals by a small number of nurses or physicians specifically trained on the protocol and study logistics, including standardized assessment of the Bishop score [27]. Written informed consent was obtained from each patient before any study-related activity which was not part of routine care was undertaken, including the performance of diagnostic procedures to determine eligibility. The consent allows for publication of the study data.

Eligible patients were healthy primiparous women, 18–40 years old with a singleton pregnancy, at ≥40 weeks gestation, as defined by the date of the last menstrual period. Inclusion criteria included a Bishop score ≤4, intact membranes, vertex presentation of the fetus, < 8 uterine contractions per hour, a reactive fetal nonstress test, a pre-pregnancy BMI ≤29 and a weight gain ≤18 kg during pregnancy. Exclusion criteria included hemoglobin <8.5 gm/dL, current diagnosis of hypertension, preeclampsia, weight <50 kg, planned and/or prior caesarean delivery or prior classical uterine incision, malpresentation of fetus, prolapsed umbilical cord, known fetal anomaly, or intrauterine growth restriction.

### Study design

Eligible subjects were admitted 24 h prior to planned induction and assigned to receive serelaxin or matching diluent (placebo). Block randomisation was used for Part A with a ratio of 4:2 (serelaxin:placebo) per dose group and simple randomisation with a 1:1 ratio was used for Part B; no stratification variables were implemented. The randomisation scheme was generated by a contract research organization, Health Decisions Ltd. (Abingdon, UK), by personnel not involved in day-to-day conduct or monitoring of the study. Sites received randomisation numbers via secure web access centrally managed by Health Decisions. Upon subject randomisation, regional central pharmacies independent of the clinics and hospitals at which subjects were enrolled prepared study drug (provided by Corthera) and provided it in identical syringes labeled in blinded fashion to the study sites. Investigators, site personnel involved in treating and assessing patients, study operations personnel, including study monitors and medical monitors, and study subjects were blinded to treatment assignments.

The study was conducted in two parts:

- Part A was a multi-center, randomized, double-blind, placebo-controlled, sequential dose escalation study evaluating the safety of 24-h continuous IV serelaxin at 7.5, 25, and 75 μg/kg/day, starting with the lowest dose. Study drug was infused using a syringe pump with infused volumes identical among all serelaxin doses and placebo to prevent unblinding. Doses were selected based on the safety profile and pharmacokinetic parameters observed in pregnant monkey studies [28–30], as well as in previously completed clinical trials in healthy volunteers or in other indications [26, 31]. Eighteen subjects were planned to be treated in cohorts of 6 and randomly assigned to receive serelaxin or placebo in a 4:2 ratio, respectively, in each cohort. Escalation to the next dose cohort occurred once maternal, fetal and neonatal safety data, including the one week postpartum assessments, were reviewed in blinded fashion by the study’s medical monitors and found to be acceptable. Safety data evaluated included maternal, fetal and neonatal adverse events (AEs), vital signs, laboratory findings and physical examination. Dose escalation was not to occur if new, clinically significant findings indicating increased maternal, fetal or neonatal risk were observed. The highest safe dose of serelaxin (maximum tolerated dose [MTD]) was to be further studied for efficacy, as well as safety, in Part B.
- Part B assessed the safety and efficacy of the MTD of serelaxin and was a multi-center, randomized, double-blind, placebo-controlled, parallel-group study of 50 subjects randomized in a 1:1 ratio to serelaxin or to placebo. In both Parts A and B of the study, dosing was to be discontinued for onset of active labour, uterine hyperstimulation and abnormal fetal heart rate, or spontaneous rupture of membranes.

Because the rate of cervical ripening potentially induced by serelaxin was unknown, three time points within the 24 h treatment period were specified and the primary efficacy endpoint was change from baseline in Bishop score at 6, 12 and 24 h (or end of study drug administration). As a pilot study, this trial included multiple exploratory secondary efficacy endpoints, including proportion of subjects with Bishop score change >3.0, time to full dilation (>10 cm), time to delivery (vaginal or C-section), incidence of vaginal deliveries and spontaneous labour, and time to onset of active labour, were also collected in this study. The 24-h changes from baseline in individual components of the Bishop score were calculated post-hoc. The sample sizes in Part A were selected empirically. The sample size estimates for Part B were based on available data from previous trials of post-date pregnant women [24, 32, 33]. A mean baseline Bishop score of three and a mean change of three in the placebo group were estimated. Since there is no previous clinical experience with systemically administered serelaxin in cervical ripening, an estimated mean change in the active group of six, achievable by prostaglandins [32], was used. Thus a sample size of 25 per group in Part B has an 83 % power to detect a difference of 3.0 in Bishop score between groups in Part B with an alpha of 0.05. This sample size was also estimated empirically to allow observation of differences between serelaxin and placebo on a number of clinically relevant endpoints.

### Safety

Maternal safety was assessed by monitoring for AEs, physical examination, ECG and vital signs at screening, baseline, serially during dosing and through 24 h post-dosing and at 2 days, 1 week and 4 weeks post-partum. Clinical chemistry and hematology were assessed at baseline, 12 and 24 h following initiation of dosing, and 2 days, 1 week and 4 weeks postpartum. Fetal heart rate was monitored pre-dose and hourly from the start of dosing to 24 h and hourly from the start of active labour until delivery. Non-stress testing was performed pre-dose and at 12 and 24 h during dosing. Neonatal safety was assessed by 1 and 5-min Apgar scores, admission to a neonatal intensive care unit (NICU) and vital signs at delivery, 2 days, 1 week and 4 weeks post-partum. Intensity of AEs was categorized by investigators using the following definitions:

- Mild: Usually transient requiring no special treatment; does not interfere with usual status or activities; awareness of event but easily tolerated
- Moderate: May be ameliorated by simple therapeutic measures; may cause enough discomfort to interfere with usual activities
- Severe: Causes inability to perform usual activities, requires close monitoring and/or intervention
- Very severe: Significantly debilitating or incapacitating despite symptomatic therapy, requires immediate intervention or emergency treatment, may be life-threatening

Serum serelaxin was measured in subjects prior to treatment and at 4, 12, and 24 h after initiation of dosing, 24 h after the end of dosing and in cord blood at delivery. Anti-serelaxin antibodies were measured in serum samples collected 1 and 4 weeks after delivery in subjects and in neonates.

### Statistical analysis

For efficacy endpoint analysis, all subjects randomized to the placebo groups in Parts A and B were pooled (“pooled placebo”) and all subjects receiving serelaxin in Part B were pooled with subjects in Part A receiving the same dose (“pooled MTD”). Because of the exploratory nature of the study, which was intended to aid in endpoint identification and sample size estimates for a subsequent larger study, this pooling was reasonable to slightly increase the power to detect differences. To explore the efficacy of a longer duration of dosing for advancement in Bishop score, a “Per Protocol” (PP) population receiving ≥18 h of study drug was pre-specified. For analysis of safety, all randomized subjects receiving any amount of study drug were included.

Data analysis was conducted by SciAn Services (Toronto, Canada), a contracted data management organization. The database was locked prior to unblinding and no changes were made to the database after unblinding. Wilcoxon rank-sum test was used to assess the difference in change in Bishop score. Student’s t-test or Chi-square test assessed differences in secondary end points. A *p* value <0.05 was considered significant.

For hematology, chemistry, vital signs, physical examination and ECGs, summary statistics were provided for baseline and changes from baseline at each time point by treatment group.

Maternal AEs were recorded according to time of onset, i.e. dosing period (0–24 h), 24-h post-infusion (24–48 h) and follow-up (>48 h). AEs reported in fetuses and in neonates were summarized by treatment group. AEs were coded using the standardized MedDRA dictionary.

### Relaxin and anti-relaxin antibody assays

Serum serelaxin was measured in an ELISA validated for measurement of serelaxin in human serum (sensitivity 96 pg/mL) and according to Good Laboratory Practices (GLP). Because serelaxin is identical to endogenous relaxin-2, both are recognized by the assay and results are presented for “serelaxin and relaxin”.

A GLP screening assay validated for measurement of serum antibodies to human serelaxin was used. If positive, samples were to be titered and checked for specificity by inhibition of binding with serelaxin.

## Results

**Fig. 1:**
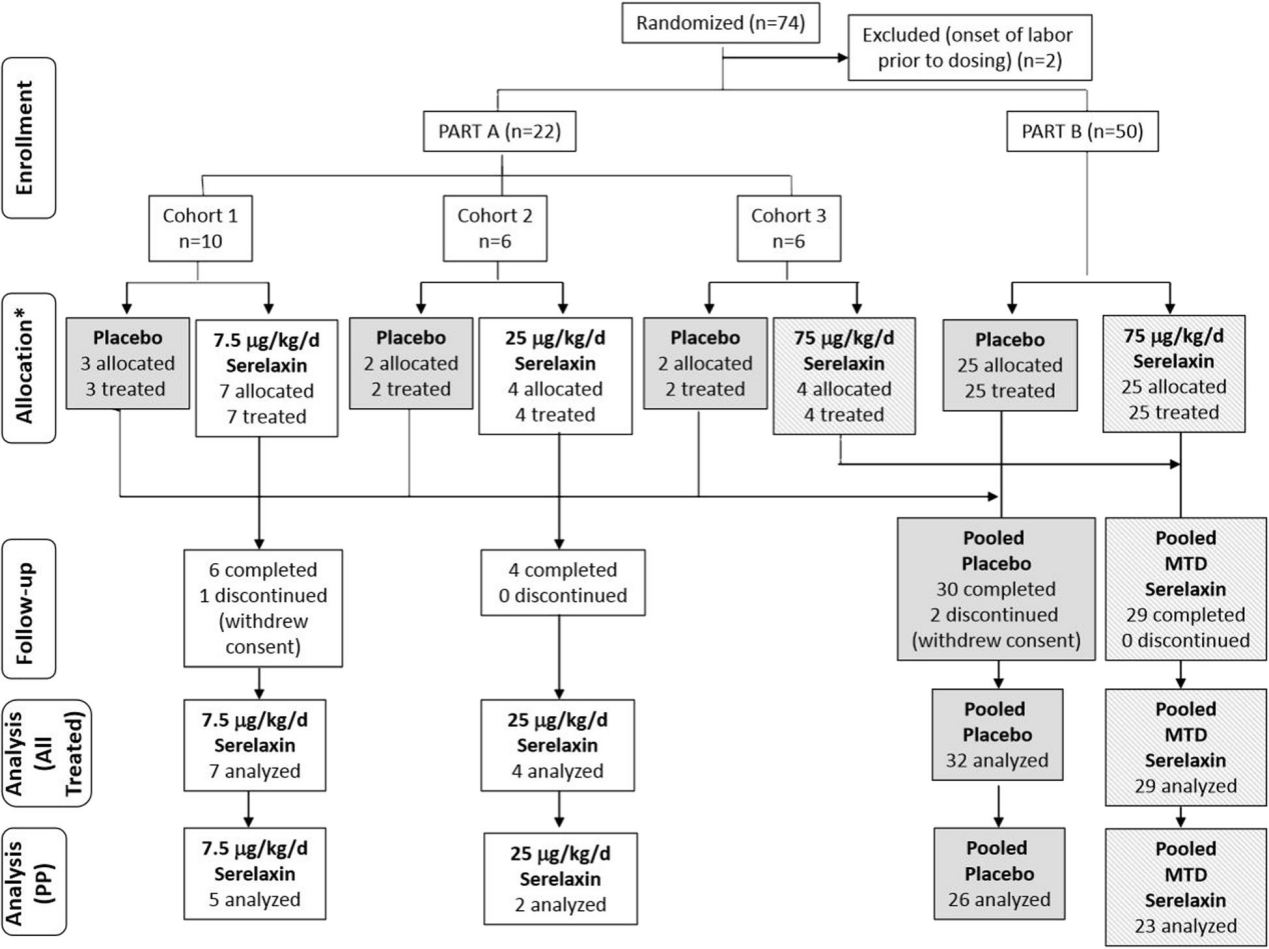
Trial profile (CONSORT 2010). *PP* Per protocol (received ≥18 h of study drug infusion). *All subjects were treated as allocated

A total of 74 subjects were randomized (Fig. 1). Two subjects went into spontaneous labour post-randomisation before treatment initiation and are excluded from all analyses. Seventy-two subjects were treated as randomized (“All Treated” population), 40 with serelaxin and 32 with placebo. Demographics were similar among subjects randomized to the two groups. Overall, the average age of the study population was 24 years with means of a BMI of 26 kg/m^2^, a pre-pregnancy weight of 60 kg, were at 40.6 weeks’ gestation, 85 % were Caucasian and 15 % were Asian. The average Bishop score was 2.1 ± 1.5 (mean ± SD) at both screening and at the pre-dose assessment.

Study drug was stopped prior to 24 h due to labour onset in 4 (13 %) and eight subjects (20 %) in the placebo and serelaxin groups, respectively. Drug was also discontinued due to rupture of membranes in one subject in each of the placebo (6 %) and serelaxin (5 %) groups. Fifty-six of the 72 subjects (78 %) received ≥18 h of study drug infusion (PP population).

Part A: Six subjects were randomized to the first cohort of Part A and an additional 4 were enrolled during the safety assessment period (Fig. 1). Therefore, in the first cohort, seven subjects received 7.5 μg/kg/day serelaxin and three subjects received placebo. The maternal, fetal and neonatal safety data, including AEs, vital signs, laboratory findings and physical examination, were found to be acceptable, so six subjects were randomized to the second cohort, receiving 25 μg/kg/day relaxin (*n* = 4) or placebo (*n* = 2). Once safety in these subjects was affirmed, six subjects were dosed with 75 μg/kg/day serelaxin (*n* = 4) or placebo (*n* = 2) in the third cohort. Based on the acceptable safety data from the 22 subjects in Part A, including similar types and distribution of AEs between groups and few AEs occurring in more than one individual, the 75 μg/kg/day serelaxin dose was determined to be the MTD and selected for study in Part B.

Part B: Fifty patients were enrolled in Part B. Serelaxin-treated subjects in Part B (*n* = 25) were pooled with subjects in Part A who received the same serelaxin dose of 75 μg/k/day (*n* = 4) for a total of 29 subjects in the pooled MTD group, and all subjects receiving placebo in Parts A (*n* = 7) and B (*n* = 25) were pooled for a total of 32 subjects in the pooled placebo group (Fig. 1). The mean treatment duration (mean ± SD) was similar in the serelaxin (22.3 ± 4.2 h) and placebo groups (21.4 ± 6.1 h). Baseline characteristics of subjects enrolled in the pooled MTD and placebo groups were similar. The average ages were 24 and 25 years, with a BMI of 26 and 27 kg/m2, respectively, and both groups were at an average of 40.6 weeks’ gestation. Ninety and 91 % were Caucasian in the pooled MTD and placebo groups, respectively, and 3 % were Asian in both groups.

The average Bishop score (mean ± SD) at baseline in the All Treated population was 2.2 ± 1.3 vs. 1.9 ± 1.6 in the pooled MTD and pooled placebo groups, respectively (*p* = NS) (Table 2). There were no statistically significant differences between groups in changes from baseline in Bishop score at any time point or when measured immediately prior to cessation of dosing (Table 1). Changes from baseline in Bishop score at 24 h in the All Treated population were 4.19 ± 1.9 and 3.26 ± 2.26 in the pooled placebo and serelaxin groups, respectively (*p* = 0.2507). In the PP population, the mean baseline Bishop score measured 2.1 ± 1.3 in the pooled placebo group and 1.6 ± 1.6 in the pooled MTD group (*p* = NS). At the 12 h time point, the increase from baseline in Bishop score was larger in the pooled placebo than in the pooled MTD group (*p* = 0.0418). No other differences between the two groups were observed. A post-hoc analysis of the changes from baseline in the individual components of the Bishop score revealed no significant differences between the pooled placebo and MTD groups in any of the components (Table 2). None of the secondary efficacy endpoints showed differences between the pooled placebo and pooled MTD groups (Table 3).

**Table 1:** Mean changes from baseline in bishop score in the all treated and per protocol (PP) populations

	Mean ± SD					
	Active (μg/kg/day)					
	Part A			Part B	Pooled MTD^a^	Pooled Placebo^a^
	7.5	25	75	75	75	Placebo
All Treated population						
N	7	4	4	25	29	32
Baseline	2.6 ± 1.6	2.5 ± 1.9	2.5 ± 1.9	2.2 ± 1.2	2.2 ± 1.3	1.9 ± 1.6
Change from Baseline:						
6 h	1.29 ± 0.95	1.25 ± 0.96	1.50 ± 1.91	1.56 ± 2.20	1.55 ± 2.13	1.77 ± 1.45
12 h	2.43 ± 1.13	2.25 ± 1.71	2.00 ± 1.63	2.13 ± 2.11	2.11 ± 2.02	2.64 ± 1.64
24 h	3.20 ± 0.84	3.00 ± 4.24	2.50 ± 1.91	3.42 ± 2.34	3.26 ± 2.26	4.19 ± 1.90
Last observation^b^	3.43 ± 0.79	2.75 ± 2.50	2.50 ± 1.91	3.76 ± 2.57	3.59 ± 2.50	4.03 ± 1.90
PP population						
N	5	2	4	19	23	26
Baseline	3.0 ± 1.7	2.0 ± 2.8	2.5 ± 1.9	2.0 ± 1.2	2.1 ± 1.3	1.6 ± 1.6
Change from Baseline:						
6 h	1.00 ± 0.71	1.00 ± 1.41	1.50 ± 1.91	0.84 ± 1.21	0.96 ± 1.33	1.65 ± 1.38
12 h ^c^	1.80 ± 0.45	2.00 ± 2.83	2.00 ± 1.63	1.63 ± 1.67	1.70 ± 1.64	2.62 ± 1.60
24 h	3.20 ± 0.84	3.00 ± 4.24	2.50 ± 1.91	3.42 ± 2.34	3.26 ± 2.26	4.19 ± 1.90
Last observation^b^	3.20 ± 0.84	3.00 ± 4.24	2.50 ± 1.91	3.42 ± 2.34	3.26 ± 2.26	4.19 ± 1.90

The Bishop score ranges from 0 (absence of any cervical changes) to a maximum of 13, representing a cervix that is dilated (>5 cm), effaced (>80 %), soft, at a +1 or +2 station, and anteriorly positioned. ^26^ If the total Bishop score was calculated >13, then ‘13’ was used. Change was calculated as Bishop score at time point – Bishop score at baseline. All Treated population includes all patients treated with any amount of study drug. Per protocol (PP) population includes all patients treated for at least 18 h of study drug

^a^Pooled Maximum Tolerated Dose (MTD) group includes subjects who were administered the 75 μg/kg/day dose in Parts A and B of the study. Pooled placebo group includes all subjects who received placebo in Parts A and B

^b^Last observation was defined as the last available value observed during dosing

^c^Difference between the pooled MTD and pooled placebo groups at 12 h, *p* = 0.042 by Wilcoxon Rank Sum test. No other differences were significant

**Table 2:** Mean changes from baseline in components of the bishop score at 24 h

	Mean ± SD	
	Pooled MTD^a^ *n* = 26	Pooled Placebo^a^ *n* = 23
Cervical Dilatation (cm)	0.63 ± 0.81	1.35 ± 1.21
Effacement (%)	0.43 ± 0.59	0.65 ± 0.75
Station	0.50 ± 1.45	0.44 ± 0.71
Consistency	0.83 ± 0.78	0.92 ± 0.63
Position of Cervix	0.83 ± 0.83	1.19 ± 0.69

Mean changes from baseline in the individual components of the Bishop score at 24 h were calculated post-hoc in all patients receiving 24 h of study drug with Bishop scores available at baseline and 24 h

^a^Pooled Maximum Tolerated Dose (MTD) group includes subjects who were administered the 75 μg/kg/day dose in Parts A and B of the study. Pooled placebo group includes subjects who received placebo in Parts A and B

**Table 3:** Secondary efficacy endpoints in the all treated population

	Pooled MTD^a^ (*n* = 29)	Pooled Placebo^a^ (*n* = 32)	*p* value
Proportion of Subjects with 24 h Change from Baseline in Bishop Score >3^b^	13/23 (56.5 %)	18/26 (69.2 %)	0.36
Incidence of Spontaneous Labour^d^	12 / 29 (41.4 %)	20 / 32 (62.5 %)	0.12
Incidence of Vaginal Deliveries^d^	20 / 29 (69.0 %)	27 / 32 (84.4 %)	0.15
Time to Vaginal Delivery, Mean ± SD^c^	62.3 ± 40.2 h	54.3 ± 29.3 h	0.64
Time to Delivery (Vaginal + C-section), Mean ± SD^c^	66.8 ± 39.6 h	54.5 ± 30.1 h	0.25
Time to Complete Dilation (10 cm), Mean ± SD^c^	57.5 ± 39.5 h	52.7 ± 26.2 h	1.00
Time to Onset of Active Labour^e^, Mean ± SD^c^	53.2 ± 36.3 h	45.8 ± 29.9 h	0.41

^a^Pooled Maximum Tolerated Dose (MTD) group includes subjects who were administered the 75 μg/kg/day dose in Parts A and B of the study. Pooled placebo group includes all subjects who received placebo in Parts A and B

^b^Subjects included have Bishop scores available at both baseline and 24 h

^c^p value was calculated using the Wilcoxon Rank-sum test

^d^p value was calculated using the Chi-square test

^e^Active labour was defined as having 35–45 s contractions every 3 min with 4 cm dilation

The treatment groups showed similar chemistry and hematology characteristics. Serum creatinine and BUN trended lower in the MTD group than in the placebo group at the 12 and 24 h time points; none of the differences were statistically significant except for lower BUN in the MTD group at the 24 h measurement (*p* = 0.02). Mean decreases from baseline in systolic blood pressure (SBP) during dosing were consistently slightly greater but not statistically different in the MTD group compared to the placebo group at all time points after 10 min. No consistent changes in heart rate were noted in study subjects or fetuses, either during or post-dosing.

**Fig. 2:**
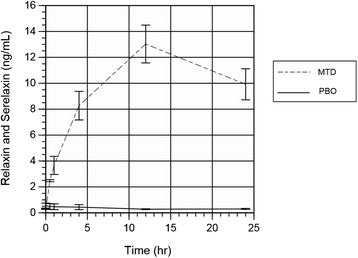
Plasma concentrations of relaxin and serelaxin. Plasma levels of relaxin + serelaxin (mean ± SEM), measured at baseline (0) and at 6, 12, and 24 h from the start of study drug administration in primiparous women ≥40 weeks of pregnancy in the pooled placebo and pooled MTD serelaxin groups. In the placebo group, only endogenous relaxin-2 is measured, while in the pooled MTD group, both serelaxin and endogenous relaxin-2 are detected

Serum serelaxin + relaxin concentration (mean ± SEM) in the pooled MTD group was 8.27 ± 1.10 ng/mL at the 4-h and 13.03 ± 1.49 ng/mL at the 12-h time point (Fig. 2). Levels in the placebo group reflecting endogenous relaxin at these time points were 0.44 ± 0.19 and 0.27 ± 0.03 ng/mL, respectively. Levels were below the detection limit of the assay in the majority of cord blood. No anti-relaxin antibodies were detectable in any of the subjects or neonates either at 1 week or 4 weeks post-partum.

### Safety

Safety was assessed in all subjects receiving any amount of study drug (*n* = 32 in the pooled placebo group and *n* = 40 in the pooled serelaxin group). Serelaxin was well tolerated in subjects, fetuses and neonates; AEs were generally balanced between treatment groups. Sixteen subjects, 8 (20.0 %) and 8 (25.0 %) in the serelaxin (all doses) and placebo groups, respectively, had 19 AEs during the 24-h dosing period. The most common maternal AE during dosing was tachycardia of mild severity (Table 4). AEs occurred in slightly more subjects in the pooled placebo (12 [38 %]) than pooled serelaxin (9 [23 %]) group during the 24 h post-infusion period, as well as during the follow up period (17 [53 %] and 19 [48 %] in the placebo and serelaxin groups, respectively).

**Table 4:** Maternal adverse events

Observation Period Adverse Event Preferred Term	Number (%) of Subjects	
	Pooled Placebo (*n* = 32)^a^	Pooled Serelaxin (*n* = 40)^a^
Study Drug Dosing		
Tachycardia	4 (12.5)	2 (5.0)
24-h Post-infusion		
Amniotic fluid decreased	3 (9.4)	0 (0.0)
Labour complication	3 (9.4)	0 (0.0)
Post-partum vaginal laceration	3 (9.4)	2 (5.0)
Uterine cervical laceration during labour	4 (12.5)	2 (5.0)
Uterine hypotonus	2 (6.3)	3 (7.5)
>48-h Post-infusion (follow-up)		
Amniotic fluid decreased	4 (12.5)	3 (7.5)
Uterine cervical laceration during labour	6 (18.8)	4 (10.0)
Uterine hypotonus	2 (6.3)	3 (7.5)
Uterine hemorrhage	3 (9.4)	0 (0.0)

Listed are all adverse events occurring in >5 % in either arm during study drug dosing, within the 24-h post-infusion period and within the follow up period

^a^The pooled placebo group includes all subjects from Parts A and B treated with placebo and the pooled serelaxin group includes all subjects from Parts A and B treated with any dose of serelaxin

There were no maternal, fetal or neonatal deaths in the study. One maternal serious AE (SAE) was reported, cephalopelvic disproportion of moderate severity in a subject receiving placebo, occurring during labour and delivery. A C-section was performed and the subject recovered.

AEs occurred in 16 fetuses, 9 (22.5 %) in the serelaxin and 7 (21.9 %) in placebo groups and were balanced between groups (Table 5). SAEs (all considered unrelated to study drug) were reported in three fetuses: acute fetal distress of moderate severity in the placebo group and two SAEs of fetal hypoxia of moderate severity in the serelaxin group, neither of which required action or medication; all fetuses recovered. All fetuses had reactive results at 12 and 24 h, with the majority having reactive results within the first 20 min and all within 40 min.

**Table 5:** Fetal and neonatal adverse events in the all treated population

Adverse Event Preferred Term	Pooled Placebo (*n* = 32)^a^	Pooled Serelaxin (*n* = 40)^a^
	Number (%) of Fetuses	
Tachycardia	2 (6.3)	1 (2.5)
Hypoxia	3 (9.4)	3 (7.5)
	Number (%) of Neonates	
Conjunctivitis	4 (12.5)	1 (2.5)
Regurgitation of food	1 (3.1)	3 (7.5)
Cerebral ischemia	8 (25.0)	3 (7.5)
Torticollis	4 (12.5)	2 (5.0)
Hypertonia	3 (8.4)	0 (0.0)
Hypoglycemia	3 (9.4)	2 (5.0)
Jaundice	2 (6.3)	5 (12.5)
Neonatal hypoxia	7 (21.9)	7 (17.5)
Neonatal agitation	7 (21.9)	4 (10.0)
Hypotonia	6 (18.8)	6 (15.0)
Neonatal asphyxia	6 (18.8)	4 (10.0)
Poor weight gain	1 (3.1)	3 (7.5)
Postmature baby	7 (21.9)	1 (2.5)
Umbilical cord around neck	5 (15.6)	3 (7.5)
Dermatitis diaper	3 (9.4)	1 (2.5)

Listed are fetal and neonatal AEs occurring in >5 % in either arm

^a^The pooled placebo group includes all fetuses or neonates from Parts A and B treated with placebo and the pooled serelaxin group includes all subjects from Parts A and B treated with any dose of serelaxin

AEs were reported in 39 neonates, 19 (47.5 %) in the serelaxin and 20 (62.5 %) in the placebo groups. The most common AE was cerebral ischemia (8/32 [25 %] in the placebo group and 3/40 [7.5 %] in the serelaxin group) (Table 5). Eleven SAEs (all determined to be unrelated to study drug) were reported in three neonates (9.4 %) in the placebo and 5 (12.5 %) in the serelaxin group. All neonates recovered from the SAEs, except for macrostomia, which was ongoing at the end of the study. Average Apgar scores were in the normal range, ≥7, for all neonates. Four NICU admissions occurred in each treatment group. At 1 week, two neonates in the placebo group remained in the NICU and at 4 weeks, one remained.

## Discussion

This study was the first to test the ability of serelaxin systemically administered at the end of pregnancy to ripen the cervix. Unlike the topical modes of administration used in previous clinical trials [20–24], IV serelaxin ensured adequate exposure of the cervix at a pharmacological dose and provided an antepartum surge of serelaxin of 20-30-fold higher than endogenous relaxin. The results indicated that while serelaxin was well tolerated, it did not enhance cervical ripening or affect relevant clinical parameters, including time to active labour, frequency of spontaneous labour or time to delivery. Because the results of this study were negative and serelaxin was being developed by the study sponsor for another therapeutic indication [34–36], publication of these data was not a priority and considerable time has elapsed since the study concluded. There was no embargo on publication and the results were presented at a meeting (5^th^ International Conference on Relaxin and Relaxin-Related Peptides, 2008) in timely manner and published in brief in meeting proceedings [37]. However, because there is continued interest in the potential ability of serelaxin to cause cervical ripening [4], the authors considered these data to be relevant and worthy of publication.

The negative results in this study may be related to the natural history of relaxin, which is species-specific in patterns of expression during pregnancy. In rodents and pigs, relaxin levels increase at the end of pregnancy in an antepartum surge of 5-20-fold mid-pregnancy levels [6] and a relaxin deficiency inhibits the dispersion of cervical collagen fibers that normally occurs during this time [7–9]. In women, relaxin levels peak during the first trimester and remain elevated compared to non-pregnancy levels throughout gestation but an antepartum surge is not observed [38], perhaps indicating a difference in relaxin biology relative to cervical ripening among these species.

Studies in rodents have indicated that relaxin also has hemodynamic properties starting mid-pregnancy when levels rise in these species [5]. Serelaxin clinical trials in other indications or in healthy volunteers have demonstrated changes in systemic and renal hemodynamics [34, 39–41], indicating that these effects translate to the human. There were trends in the current trial suggesting serelaxin slightly decreased SBP, consistent with this aspect of serelaxin pharmacology. Perhaps relaxin’s major physiological role in human pregnancy relates to maternal hemodynamic adjustments, rather than cervical ripening.

However, it is also possible that an extended duration of exposure and/or a higher serelaxin dose might enhance cervical ripening. A 24-h infusion in the setting of a clinical trial was estimated to be the longest duration consistent with management of post-date pregnant women and the dose tested did achieve a pharmacologic level that was 20-30-fold higher than physiologic exposures. Therefore, it is felt that the trial design afforded a reasonable method to test the hypothesis of therapeutic serelaxin-mediated cervical ripening.

Earlier clinical studies reported positive results of topically applied porcine relaxin [20–22], suggesting that pig relaxin may be more efficacious than human relaxin in this regard. Receptor (RXFP1) binding studies using pig relaxin have not supported this hypothesis [41], and although pig relaxin does bind with higher affinity than human relaxin to the low affinity serelaxin receptor (RXFP2), to date this receptor has not been shown to be expressed in the cervix [42]. That the RXFP2 is the receptor primarily responsible for cervical ripening in the human cannot be excluded as a possibility, but serum serelaxin at the concentrations achieved in this study should have engaged and activated both RXFP1 and −2.

In summary, this study was well designed and appropriate to test the hypothesis in that it was randomized, double blind and placebo controlled. It was designed to be a safety and exploratory dose-finding study, and a safe pharmacological serelaxin dose, whose delivery and exposure was confirmed by pharmacokinetic testing, was identified. Limitations included enrollment of small numbers of subjects from a relatively large number of institutions and the inability to test longer infusions of serelaxin. Multiple efficacy endpoints were evaluated to provide preliminary evidence of efficacy and to determine appropriate endpoints for further testing in a larger phase III study. No adjustments for multiplicity were made within any of the analyses performed; however, this would not have affected the negative results.

## Conclusions

This study indicates that a 24-h exposure to an IV infusion of 75 μg/kg/day serelaxin at the end of pregnancy was well tolerated by the subjects, fetuses, and neonates, consistent with the safety profile demonstrated in other clinical trials under conditions of much greater systemic exposure [43]. Serelaxin did not advance cervical ripening or labour.

## Abbreviations

AE: Adverse event
ECG: Electrocardiogram
IV: Intravenous
MTD: Maximum tolerated dose
NICU: Neonatal intensive care
PP: Per protocol
RXFP: Relaxin family peptide receptor
SAE: Serious adverse event
SBP: Systolic blood pressure
SD: Standard deviation
SEM: Standard error of the mean
